# Nano-immunotherapy: Merging immunotherapy precision with nanomaterial delivery

**DOI:** 10.1016/j.isci.2025.112319

**Published:** 2025-03-30

**Authors:** Thu Ha Ngo, Soumya Menon, Adolfo Rivero-Müller

**Affiliations:** 1 Department of Biochemistry and Molecular Biology, Medical University of Lublin, Lublin, Poland

**Keywords:** health sciences, medicine, medical specialty, immunology, oncology, nanomaterials

## Abstract

In current landscape of cancer treatment, nanotherapy and cellular therapy stand out as promising and innovative approaches. Nanotherapy have excelled in delivering functional molecules effectively to target cancer cells, however the targetability is mostly the result of the enhanced permeability and retention effect. Meanwhile, cellular therapies such recently emerging chimeric antigen receptor (CAR)-T therapy are proficient at specifically targeting cancer cells by using engineered receptors on T cells. Yet, cellular therapies preform poor in solid tumors due to immunosuppression and cancer cell resistance to immuno-stimulation, in other words their delivery of deadly cargo is deficient. Therefore, combining nanotherapy and immunotherapy is an emerging trend, with ongoing clinical trials exploring their synergistic effects. This 2-input approach holds promise for enhancing treatment efficacy and overcoming limitations in cancer therapy. In this review, we will discuss two aspects: targetability and delivery for each individual therapy and what the combined nano-immunotherapy strategies have achieved up to now. In the last section, some future perspectives for this combination are suggested.

## Graphical abstract

**Figure:**
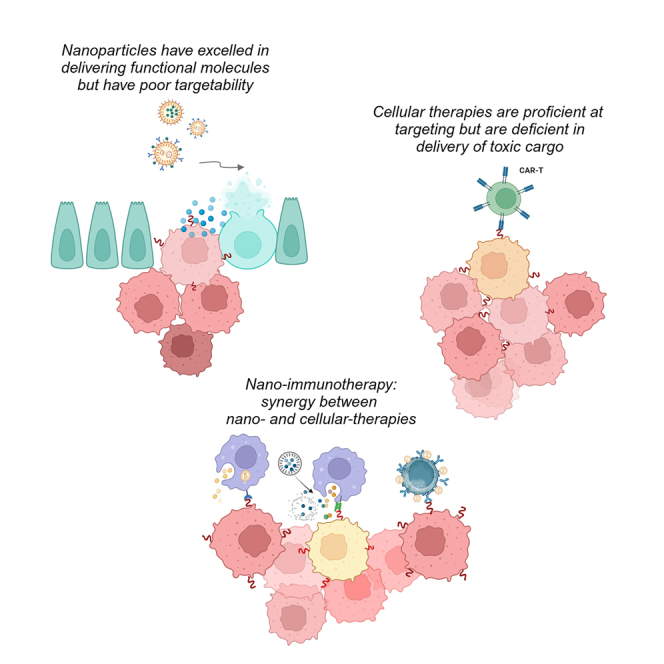

## Introduction

**Figure 1:**
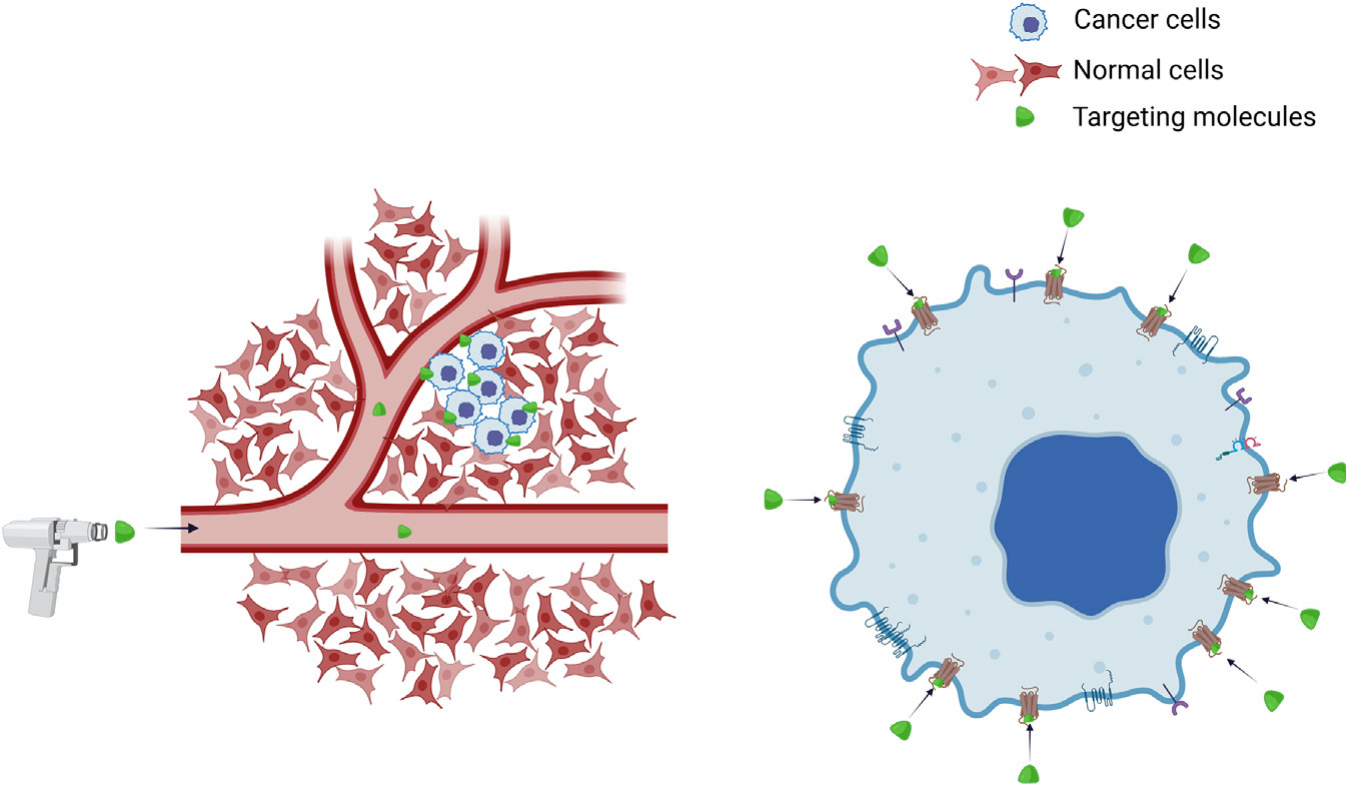
Magic bullets concept in cancer treatment refers to the ability of specifically target only cancer cells (by targeting membrane receptors/molecules expressed on cancer cells) without harming normal cells

The ability to precisely target cancer cells or tissues has been the dream of scientists since the concept of “magic bullet” was coined by Paul Ehrlich in late 1890s (Figure 1). Yet, despite more than a century of research, there are surprisingly very few truly *magic bullets*.

Current therapeutic modalities include on one hand small molecules that target signaling pathways or metabolic processes, but these suffer from solubility, stability, and lack targetability—as they affect both diseased and normal cells. On the other hand, there are antibodies and other targeting moieties that promise targetability but then rely on the immune system for action. Additional promising approaches such as nanomaterials and cellular therapies have been developed to tackle the targetability and delivery more efficiently. But, do they work as expected?

Here, we explore the advantages and limitations of the latter therapeutic modalities within the context of the most recent discoveries. Simultaneously, we suggest what potential improvements and innovations that might pave the future with a particular focus on nano-cellular therapies.

### Nanotechnology in cancer treatment

Nanoparticles (NPs) are defined by encyclopedia of pharmaceutical technology as colloidal particles ranging in diameter from 1 nm to 1,000 nm, made of various materials to be used as drug carriers.^1^ NPs have been a topic of research for seven decades, yet to-date, there is only a limited number of nano-based drugs approved by U.S. Food and Drug Administration (FDA) and/or the European Medicines Agency (EMA), summarized by Francisco Rodríguez and collaborators in 2022.^2^ In the list of approved and marketed nano-based cancer therapies, there are 3 categories.

(1) Liposomal nanodrugs made of phospholipids similar to those of the cell membrane resulting in the cellular uptake via the fusion of membranes.^3^
(2) Protein-drug conjugates are manufactured proteins with biorecognition potential e.g., antibodies, for targeting to precise molecules such as receptors, that also carry drugs, genetic material, such as DNA, RNA, or peptides. These contribute to 38% of marketed nanomedicines. Although they are not quite “particles”, such as liposomal nanocarriers and solid NPs, due to their size, protein-drug conjugates are still categorized as nano-therapies.^2^
(3) Solid NPs where the only one in the market until now is NanoTherm.^4^ NanoTherm is made of amino silane-coated iron oxide NPs that is delivered into tumors via intravenous injection and activated by a 100 kHz alternating magnetic field generator NanoActivator to produce hyperthermia at the tumor site resulting in intracellular heat stress, leading to protein misfolding and the activation of apoptosis signaling pathways.^5^

Theoretically, NPs can be engineered to carry drugs or therapeutic agents directly to a particular site in the body, maximizing treatment efficacy while minimizing side effects on healthy tissues.^6^^,^^7^ Nanomedicine, thus, holds significant promise in overcoming challenges associated with traditional small molecule drugs, particularly in the field of oncology by leveraging drug-loaded NPs to address issues, such as poor targetability, low bioavailability, and drug resistance. This approach enhances drug solubility, prolongs blood circulation half-life, and enables controlled drug release, potentially ensuring drug delivery with minimal off-target effects. Through advanced chemistry and formulation, and by coating NPs with functional molecules such as ligands or antibodies to help NPs reach the target, NPs facilitate the delivery of poorly soluble drugs, improving penetration across biological barriers and localizing delivery of higher doses.^8^^,^^9^ Yet, despite tens of thousands of research papers describing the efficiency of NPs on cancer cells *in vitro* and *in vivo*, targetability—as cell specificity and delivery—is questionable, mostly because NPs seem to naturally accumulate within tumors, as non-functionalized “naked” NPs are up-taken by cells often in equal manner. Indeed, while NPs have shown *in vitro* and *in vivo* to be able to deliver different sets of molecules to cells,^10^^,^^11^^,^^12^^,^^13^^,^^14^ it is their targetability that should be questioned. For example, several studies have reported that “naked” NPs stabilized with various polymers like polyvinyl pyrrolidone (PVP), polyethylene glycol (PEG), or polyvinyl alcohol (PVA) led to an intracellular delivery of their cargo causing cell death in *in vitro* studies, without any active targeting ligands present.^15^^,^^16^^,^^17^^,^^18^ It is clear now that delivery and targetability are two equally important aspects leading to therapeutic success in the clinic. While NPs can undoubtedly enhance drug delivery to tumors, this seems to be mostly the result of the enhanced permeability and retention (EPR) effect, in which macromolecules accumulate in solid tumors—a unique phenomenon that occurs in response to uncontrolled and disordered tumor growth leading to leaky blood vessels, where large molecules such as NPs roost in the interstitial space of the tumor, creating the illusion of targetability, contributing to the off-sides of nano-therapy and its failure in the clinic.^19^

#### Questions on targetability of NPs

As mentioned earlier, the *magic bullet* concept has inspired researchers to develop formulations for drug and vaccine delivery, among these formulations a large research effort has been focusing in NPs.^1^ Targeted therapy, one of the currently common systemic therapies (including chemo-, immuno-, endocrine-, and targeted-therapy) used for cancer patients, aims at specific molecules, varying from antigens to membrane receptors to growth factors that are uniquely or mostly (over)expressed on cancer cells.^20^ The same principle has been applied to NPs, by functionalizing solid or lipid NPs with such bio-recognizing molecules it is expected selective targeting and delivery. Yet, there are two mechanisms of action that guide them to the tumor site for the mission of targetability: active or passive.

Active targeting frequently involves the interaction between ligands and receptors or antibodies and antigens. The receptor-mediated targeting is a common strategy aiming at overexpressed receptors on the tumor cell surface to facilitate NPs binding without hampering normal cells. For example, human epidermal growth factor receptor 2 (HER2), which is overexpressed in some types of breast cancer cells, is one of the most common targets in breast cancer treatment.^21^ With this in mind, Mingyang Li and collaborators functionalized silica NPs with 50–180 molecules of the fragment antigen-binding region of an antibody (Fab) against HER2 per NP, showing binding to HER2 and a quick response in HER2-positive cells.^22^ Yet, this is one of the very few examples where binding has been quantitatively measured and where the protein coating, known as “corona”, formation was analyzed. In most cases, however, “naked” and functionalized NPs using ligands like folate, transferrin, and hyaluronic acid are internalized often at equal or higher rates by cells.^23^^,^^24^ The poor targetability is caused by nonspecific binding to plasma membranes, similar to what has been observed with antibodies.^25^^,^^26^^,^^27^^,^^28^

Contrary to active targetability, the spontaneous accumulation at tumor sites and the interaction with target cells without the presence of a specific targeting moiety is typical passive targetability. The EPR effect has been suggested as the most critical passive targeting mechanism for liposomal NPs while it seems to be a size dependent process for solid NPs as suggested by some reports.^29^^,^^30^ Though, controversy exists about the EPR effect as an advantageous or a disadvantageous characteristic to enhance the treatment efficiency of NPs.^31^ For example, the EPR is also attractive to proteins or factors produced by immunosuppressive cells within the tumor microenvironment (TME), some of them are known to reduce or block the interaction between NPs and tumor cells.^32^ To date, results from preclinical trials show that nanomedicines accumulate differently in the EPR of various cancer types.^33^ The EPR is known to be a main problem for antibody-based therapies as well, because they seem to “target” via the specific interaction between antigen-antibody but in practice, due to their nano size, they just accumulate in tumors due to EPR as reviewed by Hannes Ausserwöger and collaborators.^25^

**Figure 2:**
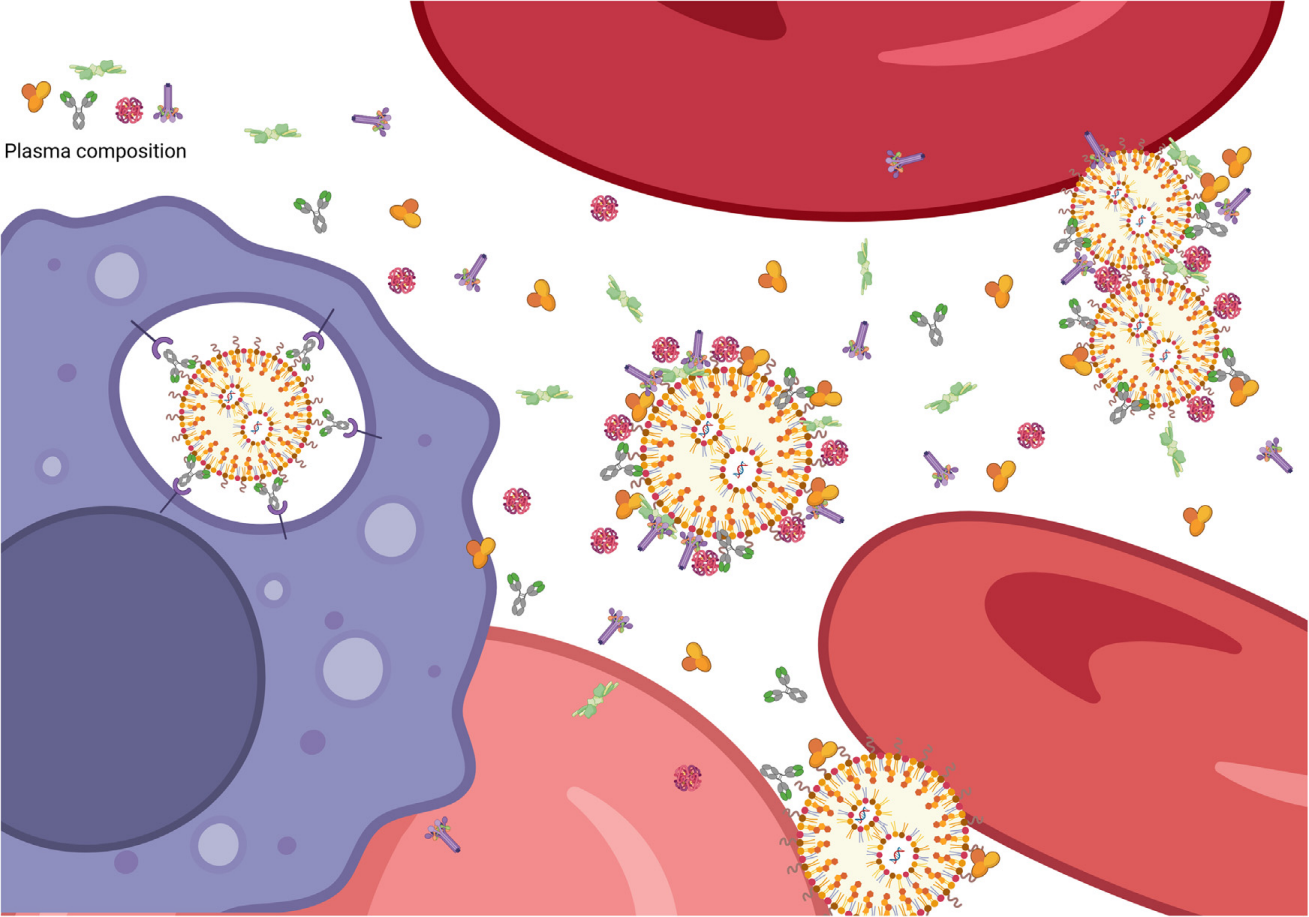
Illustrates the interaction of NPs with biological fluids The plasma proteome consists of proteins carrying out various functions in circulation, such as antibodies, growth factors, albumin, coagulation factors, fibrinolytic proteins … and can bind to nanoparticles leading to corona formation. In some cases, the nanoparticle may be opsonized resulting in engulfment by macrophages (purple cell) or loss of function.

The main problem of passive accumulation of NPs is that it occurs not only in the TME but also at physiological barriers belonging to the immune system such as liver and spleen which are responsible for removing foreign particles from circulation and coincidently becoming the center of off-target toxicity.^34^^,^^35^^,^^36^ In addition, once NPs encounter biological fluids, the structural preservation of NPs is challenged. These fluids (mostly plasma in this case) contain multi components, such as ions, proteins, hormones, growth factors that affect to the structure-function relationship of NPs—the so called “corona formation” signifying the nano-bio interaction between NPs and plasma components caused by the size, shape, and surface properties of NPs (Figure 2). The corona formation seems unpredictable and how this new coat affects interactions with cells is less understood or controllable.^37^^,^^38^^,^^39^^,^^40^ The components of such corona, which differ even between the serums of different species toward the same NPs, affect how the NPs interact with targeting and immune cells such as macrophages.^41^^,^^42^^,^^43^^,^^44^ Moreover, how the protein corona affects further ligand-receptor interactions has been rarely reported in detail.^45^^,^^46^ While the corona can block the binding of functionalized ligand or antibody on NPs to targeted receptors, it also makes these NPs more attractive to be internalized by other cell types such as THP-1 macrophages (phagocytic-differentiated macrophage-like THP-1 cells).^37^^,^^47^

Is “targeting”, by EPR or membrane interactions, true targeting (specific to cancer cells) or just near-by targeting? Unfortunately, there are only a few examples where direct interactions between ligands on NPs, before and/or after coronal formation, and the corresponding receptors on cells are reported, as most NP research has been focusing on overexpressing receptors on cells versus cells lacking such receptors.^48^^,^^49^ This lack of active targetability is likely one of the reasons for unsuccessful results in clinical trials.

### Cellular therapies—true targetability

Cellular therapy refers to the use of cells to regenerate the function of damaged tissues or cells selected or engineered to target other cell types. This is performed by transferring autologous (using the patient’s cells), syngeneic (cells from twins), or allogeneic (from another person) cells to a patient.^50^ The concept of cellular therapies is not new, but only recently have genuine breakthroughs in immunotherapy gathered pace, such as adoptive cellular therapies (ACTs), including tumor-infiltrating lymphocytes (TILs) and chimeric antigen receptor (CAR)-T cells. Immune cells (T cell and macrophages, for example) are natural seek-and-destroy biological machines with the ability to cross tissue barriers and penetrate into tissues.

TIL therapy involves the use of isolated TILs from the TME of a patient, such cells are expanded *in vitro* and finally injected back to the patient in combination with high-dose of IL-2 to increase the therapeutic effectiveness.^51^ Unfortunately, TIL therapy is still undergoing clinical trials and has not obtained approval for worldwide use due to the following difficulties: although TILs are found in the TME, successful isolation and amplification are limited due to their rare occurrence, and their ability to kill cancer cells is further affected by TME components including immunosuppressive cells and cytokines.^52^

**Figure 3:**
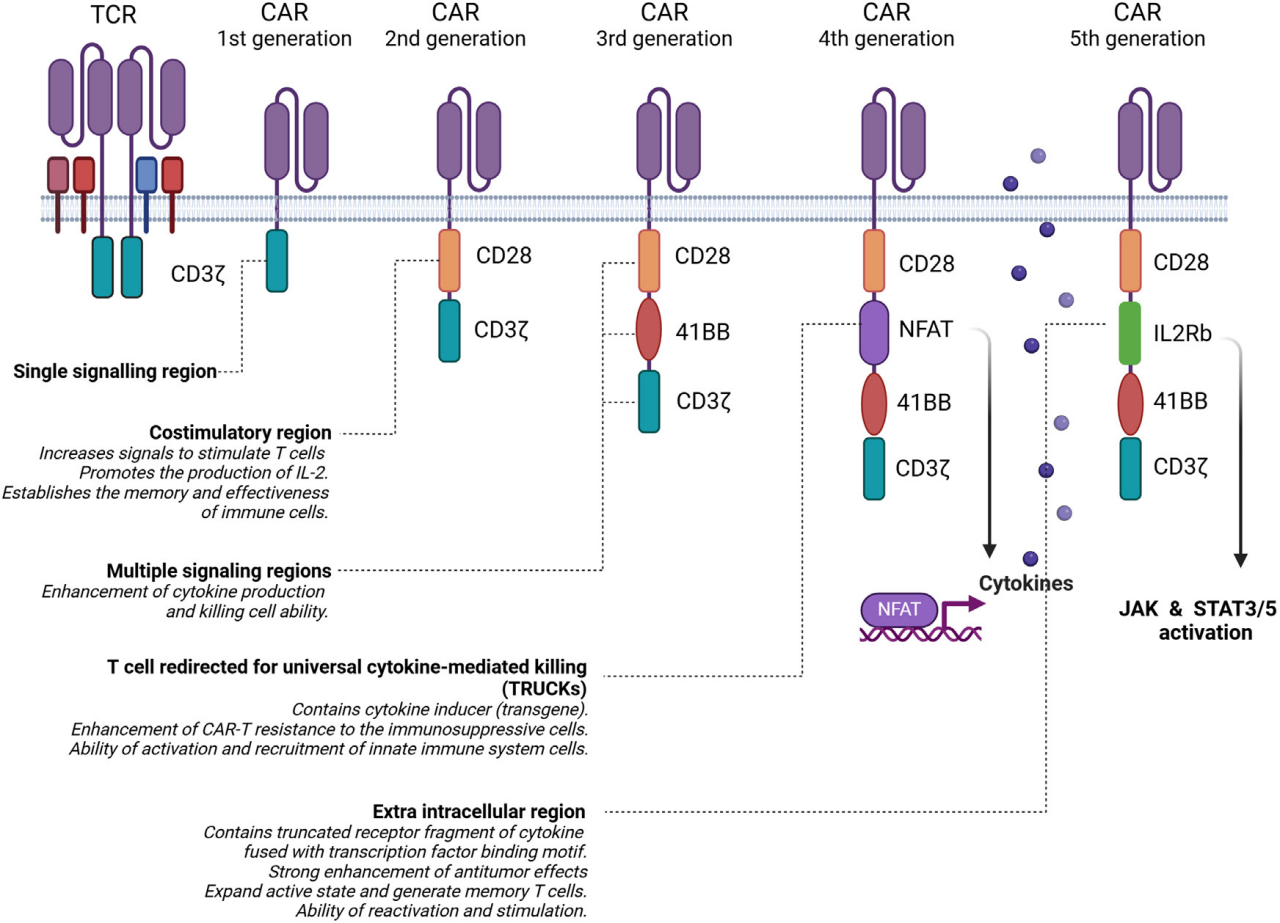
Five generations of CAR-T to-date The evolution of the engineered chimeric antigen receptor (CAR).

Instead of collecting existing immune-targeting cells with unknown potential, genetic and protein engineers have re-engineered the antibody-presenting T cell receptor (TCR) complex into a single-molecule receptor, named CAR that recognizes a specific antigen on target cells. Since 1980s when the initial establishment of CAR was introduced, there have been 5 generations based on changes of the endo-domains of three immune-receptor tyrosine-based activation motifs (ITAMs) responsible for signal transduction upon activation on T cells (Figure 3). The genetically engineered CAR-coding gene is then integrated into the genome of T cells (CAR-T) using different methods such as viral transduction, transposons or CRISPR-Cas9.^53^ In 2017, a milestone for the successful development of CAR-T therapy took place when FDA and EMA approved Kymriah for the treatment of non-Hodgkin lymphoma, where the CAR was engineered to target the cluster of differentiation (CD)-19 expressed on the surface of B cells.^54^

Similar to TIL therapy, the selection of CAR-T starts with the isolation of leukocytes from patients/donors to be enriched and separated between T CD4^+^ and T CD8^+^ in the laboratory. Then, these cells are genetically engineered before being infused back into patients.^53^ This process may bring about risks such as unwanted mutagenesis or transposon remobilization; however, the frequency seems to be insignificant.^55^ Despite the success of CAR-T cell therapy in blood cancers (lymphoma or myeloma), remaining difficulties in the transition of immune cell therapy against solid tumors exist due to either tumor heterogeneity, off-target effects, immune-tolerance, immune-suppression, or the poor penetration of CAR-T cells into solid tumors.^56^

**Figure 4:**
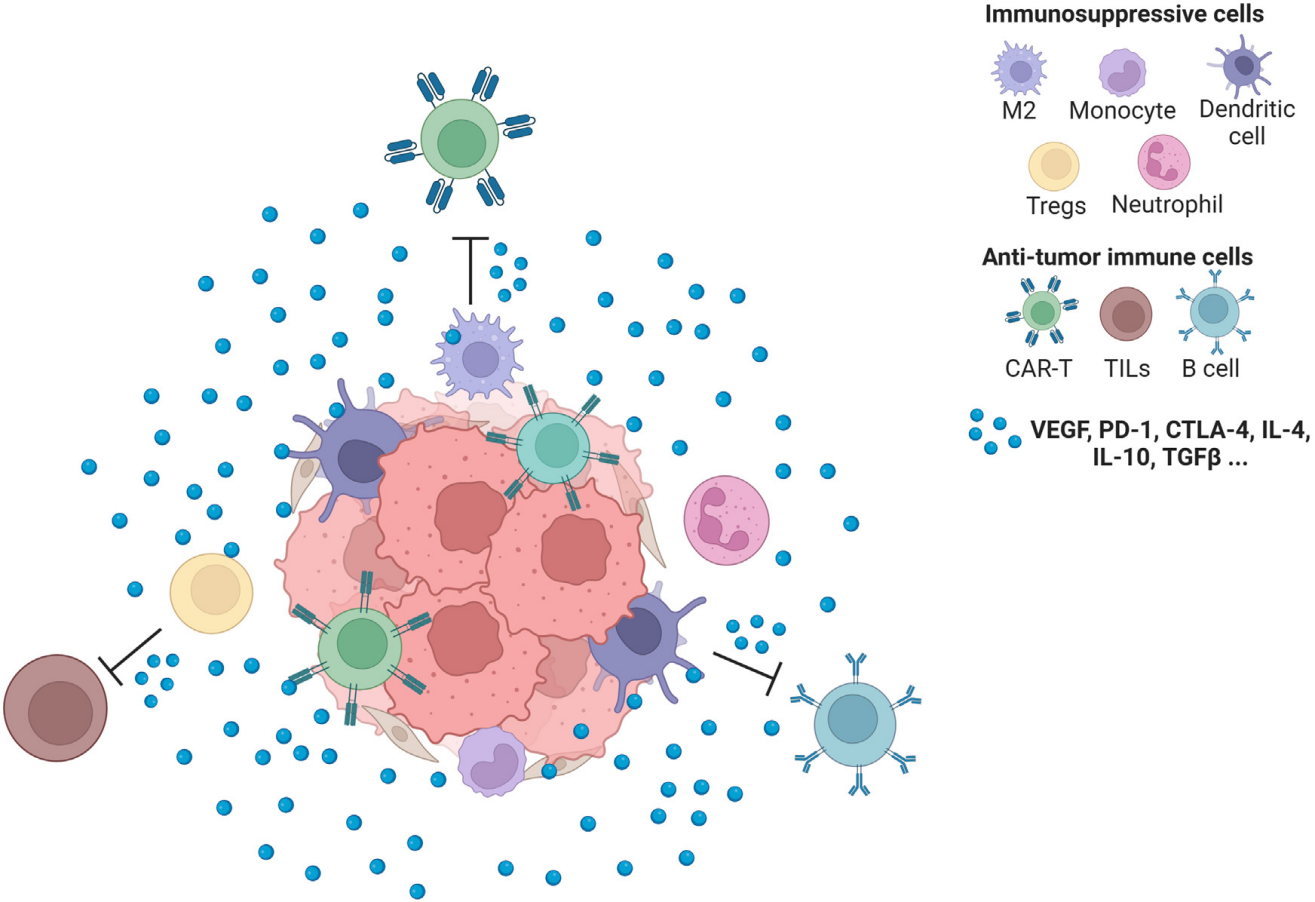
The interaction between tumor cells and other cells in TME The tumor microenvironment (TME) is a complicated ecosystem composed of cancer cells, immune cells, fibroblast, signaling molecules and the extracellular matrix. Immune cells in the TME have opposing functions: while TILs and CTLs seek out and destroy cancer cells, immunosuppressive cells secrete molecules, such as VEGF, IL-10, TGF-β, and PD-1, stimulating tumor growth and exhausting or competing with anti-tumor immune cells.

Tumors are heterogeneous in terms of their various cell types, including cancer cells, TILs, cytotoxic T lymphocytes (CTLs), cancer-associated fibroblasts (CAFs), immunosuppressive cells that secrete cytokines, chemokines, growth factors, and other molecules, and the extracellular matrix—all constituting to the TME (Figure 4).^57^ Some of the most notably immunosuppressive cells are tumor-associated macrophages (TAMs) that predominate in tumors and have the ability to transition from an anti-tumor M1-like phenotype to a pro-tumor M2-like phenotype as tumors progress.^58^ This transformation, influenced by factors such as metabolic changes, lactic acidosis, angiogenesis, and stromal remodeling, leads TAMs to engage in immunosuppression, angiogenesis, and other processes that support tumor growth. Two other main cell types contributing to the immunosuppression found in tumors are: myeloid-derived suppressor cells (MDSCs), which deplete essential amino acids (*L*-arginine and cysteine) necessary for the function of T cells, and regulatory T (Treg) cells which play a vital role in self-tolerance maintenance and can also act as an immunosuppressive barrier (a subset of CD4^+^ T cells only), inhibiting anticancer immunity in tumor-bearing hosts and impeding protective immunosurveillance against neoplasia.^58^

Moreover, the variability in antigen expression (both the number of antigens and their expression levels) by different cell types in the TME reduces the recognition potential of CAR-T cells.^59^ In addition, many of the targeted antigens are also present in other tissues and organs, as mentioned in the targetability of nanodrugs section, causing off-target immune reactions, autoimmune toxicity and an overall reduction of the efficiency of therapy in the clinic. For example, the 1^st^ generation CAR-T targeting the carcinoembryonic antigen (CEACAM5) in the gastrointestinal tract resulted in respiratory toxicity to patients due to high level of interleukin (IL)-6 secreted by CAR-T cells in the lungs, where this antigen is also found at low levels.^60^ Such immune hyperactivation in the presence of low or undetectable antigen levels results in the cytokine release syndrome (CRS), also called *cytokine storm*, where excessive cytokines production induced inflammation and damaging tissues and organs.^61^ Two interleukins, IL-1 and IL-6, are involved in CRS and can cause severe adverse events, such as fever, headache, hypotension, and even life-threatening conditions.^62^ In general, CRS is a systemic inflammatory response happening when a large number of immune cells are activated, commonly a few days after CAR-T infusion to the patients. In fact, the mechanism driving CRS is not fully understood, as in the event that a CAR-T cell binds to its specific antigen leads to an excessive release of interferon (IFN)-γ which then activates the immune cell repertoire, especially macrophages, leading to the inflammatory process that overwhelms homeostasis.^63^ CRS is also a frequent event observed in bi- or tri-specific T cell engager (BiTe or TriTE) therapies, which are recombinant antibodies composed of at least two different antibody fragments that bind to both T cell specific receptor (typically CD3) and tumor-associated antigens (TAA), thereby inducing a cytolytic synapse between T cells and malignant cells.^64^ BiTe and TriTE have shown potential in the treatment of solid tumors, as demonstrated by Rybrevant and Imdelltra, which were approved by the FDA for the treatment of lung cancer.^65^ Additionally, a number of clinical trials in the treatment of breast, gastric, bladder, prostate, and esophageal cancer, among others, are currently underway. For more information, we refer the reader to a recent in-depth review by Junjun Liu and Jianwei Zhu.^66^ Although showing promising potential due to their ability to target more than one antigen, these bi- or tri-specific antibodies have complex pharmacokinetics and short half-lives.^66^

Another characteristic contributing to the difficulty in the transition of immuno-therapy to clinical trials is tumor heterogeneity because the different cell types, including immune cells, within the tumor and TME.^57^ The most common problem is immune-suppression, mostly caused by the programmed cell death protein 1 (PD-1) expressed on the surface of both B and T lymphocytes, especially after antigen presentation, directly inhibiting TCR and CAR-T stimulation.^67^^,^^68^ PD-L1, the ligand of PD-1 receptor, is often up-regulated by malignant cells, causing the secretion of pro-inflammatory cytokines, such as IL-6, IFN-γ, and tumor necrosis factor (TNF) leading to the exhaustion of T cells resulting in immunotherapy resistance—a process called *immunoediting*.^69^ The interactions between immune cells and cancer cells are initially mediated by precursors of CAFs through cytokines and the extracellular matrix (ECM).^70^ These precursor CAFs then transform into CAFs and recruit immunosuppressive cells to the TME via transforming growth factor β (TGF-β), MCP-1, and IL-10, leading to competitive communication between immunosuppressive cells and effector cells, thereby reducing the effectiveness.^71^ Besides, the mismatch of chemokine expressions in different cell types contributes to the loosely immune-cancer cell response. For example, the imbalance between chemokine receptor CXCR3 expressed by TILs that traffic CTL to the TME and CXCL9/CXCL10 (ligands of CXCR3) up-regulated by tumor cells leads to inefficient recruitment of CTL to the tumor. Additionally, these CTLs are naturally hindered from penetrating vascular endothelium and are suppressed by inhibitory checkpoint molecules, such as PD-L1, IL-10, and TGF-β secreted by suppressive immune cells, facilitating the proliferation of cancer cells and contributing to tumor growth.^72^ It is clear that trafficking and infiltration of immune cells to the tumor present a challenge to cellular therapies. We recommend the following expert reviews on the PD-L1/PD-1 roles in cancers, as well as their potential application and challenges in immunotherapy.^73^^,^^74^^,^^75^

Since T cells seem to poorly infiltrate into solid tumors, researchers have navigated to macrophages as a new promising approach. CAR-macrophages (CAR-M) were first published by Klichinsky and colleagues in 2020 and are now undergoing phase I clinical trial (ID NCT04660929), using a similar approach to the 1^st^ generation of CAR-T targeting CD19 expressed on B cells.^76^^,^^77^ The advantages of CAR-M include the ability of macrophages to infiltrate deep into tissues, recruit lymphocytes, present antigens, as well as directly kill cancer cells by secreting cytokines and oxygen radicals.^76^^,^^78^ However, as mentioned before, IL-6 is considered as the key role in CRS pathophysiology because of its consequences, including the increase of vascular permeability caused by the activation of complement components and the secretion of cytokines caused by the differentiation of naive T cells to effector cells, naive B cells to antibody-producing cells.^79^ More importantly, monocytes which produce IL-6, are the precursors of macrophages, if using CAR-M to circumvent the disadvantages of CAR-T, could CAR-M therapy escape cytokine storm since macrophages themselves can produce IL-6? Ultimately, could a single therapy be sufficient? Or would a combination of therapies be more optimal?

### Can the combination between nano- and cellular-therapies overcome their respective limitations?

On one hand, there is nanotechnology, where delivery of small molecules, and not so small ones such as mRNA, is efficiently achievable but targeting is a severe limitation.^80^ On the other, cellular-therapies are indeed efficient at specific targetability; however, the difficulties in their transition to the clinic for solid tumors lie in the infiltration of immune cells into the tumor and the delivery of therapeutic molecules. Could T cells or macrophages, in combination with nanotherapy become the next strategy for cancer treatment? This is a rather new concept, as a search “nanotherapy” and “immunotherapy” in PubMed showed mostly results published in 2022 and 2023. Interestingly, these publications focus mostly on nanoformulations boosting immune cells on solid tumors.

So far, the following approaches have been explored to combine nanotechnology and cellular therapy.

(1) The use of nanomaterials to activate immune cells;
(2) The synergy of nano-delivery and cellular therapy;
(3) The guiding or delivery of nanoformulations by immune cells to tumor tissue;

#### NPs as activators of immune cells

T cells, important components of the adoptive immune system due to their ability to kill target cells, have been a powerful strategy in cellular therapy, although having persistent challenges in solid tumors, as mentioned earlier. T cells are normally activated when antigens are presented by antigen-presenting cells (APCs) such as dendritic cells (DCs), B cells or macrophages, via the major histocompatibility complex (MHC). These specialized cell populations provide B and T cells with at least two different stimuli: the initial signal is produced when the MHC-peptide complex on APCs binds to the clonotypic TCR. Costimulatory molecules, such as CD80/CD86 on APCs and CD28/CTLA-4 on T lymphocytes, produce the second signal and are not MHC-restricted. Based on such natural strategy, NPs have been engineered as artificial antigen-presenting “cells” (aAPCs) mimicking APCs to stimulate T cell expansion and enrichment, both *in vitro* and *in vivo*. As such, nano-aAPC made of dextran-coated paramagnetic iron oxide NPs coupled with MHC/HLA-A-0201 (an antigen of MART-1) and anti-CD28 enriched MART-1-specific CD8^+^ T cells up to 1000-fold after 14 days of treatment and expanded cell populations with a memory phenotype expressing CD45RA, CD62L, CD95, as well as longer telomeres compared to unexpanded T cells, as shown by Junya Ichikawa and collaborators investigating melanoma cell lines.^81^^,^^82^^,^^83^

#### Synergy of delivery by NPs and targeting by cellular therapy

Tumors exhibiting immunological tolerance, characterized by limited infiltration of CTLs and an immunosuppressive microenvironment, present an ongoing challenge for immune checkpoint blockade (ICB) therapy, due to their resistance to checkpoint inhibitors. ICB therapy is a promising approach in cancer immunotherapy, actively modulating the immunosuppressive TME. Therapeutic monoclonal antibodies, such as atezolizumab, nivolumab, durvalumab, and pembrolizumab, targeting the PD1/PDL1 checkpoint, have demonstrated lasting tumor regression across various cancers.^84^^,^^85^ In order to engineer an activatable ICB for targeted delivery, researchers developed a matrix metalloproteinase protein 2 (MMP-2)-activatable anti-PDL1 antibody (αPD-L1) conjugating NPs. These NPs also contain a photosensitizer which upon near-infrared (NIR) irradiation induces reactive oxygen species (ROS) production and releasing the αPD-L1, promoting an enhanced penetration of CTLs into MMP2-overexpressed tumors, resulting in notable therapeutic efficacy. Nevertheless, further optimization is warranted to expand its applicability across a wider range of solid tumors and facilitate its translation into clinical settings.^86^

A novel approach, termed *immunoswitch*, has been introduced recently that links ICB and the costimulatory signals of T cells such to enhance immune response.^87^ The development of immunoswitch particles as an NP platform for combinatorial immunotherapy is a significant advancement in cancer treatment. These particles may effectively modulate the TME by inhibiting the PD-L1 pathway on tumor cells while activating the 4-1BB pathway on CD8^+^ T cells, resulting in synergistic antitumor effects. *In vivo* studies across melanoma and colon cancer models demonstrated their efficacy, even in the absence of any foreign antigen. Importantly, immunoswitch particles enhance tumor-specific CD8^+^ T cell activation and induce changes in the endogenous T cell receptor repertoire, leading to improved recognition and efficacy against tumor antigens. This approach offers the simultaneous targeting of multiple stages of the cancer immunity cycle.^87^ Likewise, an article by Ara Sargsian and collaborators reported that small (5 nm) silver-citrate NP could “target” (NPs were injected intratumorally) murine renal adenocarcinoma (Renca) cells allografted into mice. The combination therapy between these Ag-citrate NP and anti-PD1 antibodies induce cytotoxicity by ROS production and the secretion of cytokines that in turn induced an immune response, mostly by recruitment of CD8^+^ T cells to tumor sites.^88^ In another study, the combination between BiTE (composed of anti-PD/L1 and anti-CD3) and ganetespib-loaded PEG nanoparticles improved cellular uptake and enhanced the recruitment of CD8^+^ T cells to the tumor site.^89^ Ganetespib, an inhibitor of heat shock protein 90 (HSP90), is being tested in multiple clinical trials for the treatment of solid tumors, including lung cancer (NCT01579994), ovarian cancer (NCT03783949), breast cancer (NCT01560416) showing the potential to interfere with signal transduction, apoptosis, cell cycle, which are cellular processes associated with the overexpression of HSP90.^90^

The accumulation of metabolic waste in the TME, such as lactate from glycolysis, contributes to the acidity of the TME and is considered a driver of cancer progression by promoting immunosuppression.^91^ The Warburg effect, characterized by high glucose uptake, lactate production under oxygen availability, disturbs the glycolysis of effector T cells, leading to their reduction of proliferation, cytotoxicity and cytokine production, contributing to the diminished effectiveness of immune-therapy.^92^ Therefore, reducing lactate concentration is crucial for optimizing T cell therapy. With that in mind, Zheng Cao and collaborators introduced lactate oxidase (LOx) nanocapsules, termed n(Lox), in combination with αPD-L1 therapy, showing the ability to boost T cells by leveraging hydrogen peroxide (H_2_O_2_) to recruit effective T cells toward the injury site.^93^ Furthermore, H_2_O_2_ activates T cells via IFN-γ and NF-κB signaling pathway, promoting the interaction between CXCR5 and its ligand CXCL13, and thus, facilitating T cell migration to the TME.^93^^,^^94^ The use of nLOx not only reduces lactate concentration but also releases H_2_O_2_, alleviating immunosuppressive activity and improving therapy efficiency. A similar approach to take advantage of the acidic TME has been reported by a group of scientists led by Quan Zhou. They modified iron oxide NPs (named FGR), coated with Resiquimod (R848)—an agonist of TLR—via an acid-sensitive bond.^95^ In the acidic TME, the acid-sensitive bond is hydrolyzed, releasing R848 to stimulate not only TLR to promote the maturation of dendritic cells but also effector T cells resulting in increased T cell penetration into tumors and improving immunotherapy.^96^

**Figure 5:**
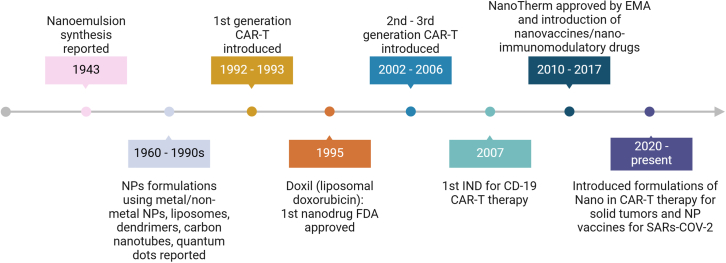
A timeline of key milestones in the developments of Nano-immunotherapy This timeline traces advancements from the early groundwork laid in the mid-20^th^ century with the reporting of nanoemulsion synthesis and the exploration of nanoparticle formulations using diverse materials, to the emergence of liposomal doxorubicin (Doxil) as the first nanodrug approved by the FDA in 1995. The late 20^th^ and early 21^st^ centuries witnessed crucial progress in both CAR-T therapy, with the introduction of 1^st^ generation CAR-T and its subsequent evolution into 2^nd^ and 3^rd^ generation CARs, and in nano-immunotherapy with the approval of Nab-paclitaxel (Abraxane) and the clinical translation of NanoTherm for cancer treatment. More recently, the convergence of nanotechnology and immunotherapy has gained momentum, marked by the first clinical trial of CD19 CAR-T therapy, the introduction of nano-immunotherapy formulations for solid tumor treatment, and the development of nanoparticle-based vaccines for SARS-CoV-2, demonstrating the expanding potential of this rapidly advancing field.^98^^,^^99^^,^^100^

Currently, most nanomaterial/immunotherapy involves the use of NPs to deliver anti-cancer drugs in synergy with antibody-targeting moieties against specific tumor types, as summarized in Table 1. We also highlight milestones in the development of nano-immunotherapy as presented in Figure 5.

**Table 1:** Ongoing combinational treatment of NPs, chemotherapeutic drugs, and immunotherapeutic agents in clinical trials

Compound Name	Cancer	NP	Payload	Immunotherapeutic agents	Mechanism of action	Phase	Clinical trial.gov ID
PRECIOUS-01	New York esophageal squamous cell carcinoma-1(NY-ESO-1)	Poly(lactic-co-glycolic acid) (PLGA) NP	NY-ESO-1 antigen peptides	Invariant natural killer T cell (iNKT)	Antigen presentation	I	NCT04751786
NBTXR3	Recurrent or metastatic head and neck squamous cell cancer	Hafnium oxide-containing NPs		Pembrolizumab (Anti PD-L1/PD-1)	Anti-tumor immune stimulation	II	NCT04862455
NBTXR3	Lung/liver metastatic	Hafnium oxide-containing NPs		Nivolumab (Anti-PD-1/PD-L1)	Immunological cell death	I/II	NCT05039632
AR160	B cell non-Hodgkin lymphoma	Nab-paclitaxel		Rituximab (Anti CD20)	Tumor-targeting via complement-mediated cytotoxicity (CMC), and antibody-dependent cellular cytotoxicity (ADCC)	I	NCT03003546
	Stage III non-small cell lung cancer (NSCLC)	Nab-paclitaxel		Durvalumab (Anti-PD-1/PD-L1)	Anti-tumor immune stimulation	I	NCT05157542
	Extramedullary myeloma (EMM)	64Cu Super paramagnetic iron oxide NP (64Cu SPION)		Ciltacabtagene autoleucel (CAR-T anti B cell maturation antigen)	*In vivo* trafficking	Ib	NCT05666700
	Triple negative-negative breast cancer	Nab-paclitaxel	BET Bromodomain Inhibitor ZEN-3694	Pembrolizumab (Anti-PD-1/PD-L1)	Anti-tumor immune stimulation	I	NCT05422794
	Malignant solid neoplasm	Pegylated Liposomal Doxorubicin Hydrochloride, Nab-paclitaxel	Capecitabine, Carboplatin, Gemcitabine Hydrochloride, Paclitaxel	Durvalumab (Anti-PD-1/PD-L1)	Anti-tumor immune stimulation	II	NCT03907475
	Abdomen or thorax	Nab-paclitaxel	Cabozantinib S-malate	Atezolizumab (Anti-PD-1/PD-L1)	Anti-tumor immune stimulation	I	NCT05092373
	Triple negative breast cancer	Nab-paclitaxel		Atezolizumab (Anti-PD-1/PD-L1)	Anti-tumor immune stimulation	II	NCT02530489
	Resectable/borderline resectable primary pancreatic cancer	Nab-paclitaxel	Gemcitabine	Durvalumab (Anti-PD-1/PD-L1), and Oleclumab (Anti-CD73)	Disrupting extracellular adenosine production and downstream immunosuppressive effects	II	NCT04940286
	Lung cancer	Nab-paclitaxel	Carboplatin, Gemcitabine, Paclitaxel, Pemetrexed	Durvalumab (Anti PD-L1/PD-1)	Anti-tumor immune stimulation	II	NCT04892953
	Advanced non-small cell lung cancer	Nab-paclitaxel	Paclitaxel, Pemetrexed, Carboplatin	Nivolumab (Anti PD-L1/PD-1), Ipilimumab (Anti-CTL-4)	Anti-tumor immune stimulation	II/III	NCT04929041
	Stage IV lung cancer	Nab-paclitaxel	Carboplatin, Fludeoxyglucose F-18, Pemetrexed	Pembrolizumab (Anti PD-L1/PD-1), Ipilimumab (Anti-CTL-4), Nivolumab (Anti-CTL-4)	Anti-tumor immune stimulation	I/II	NCT05501665
	NSCLC, brain cancer	Nab-paclitaxel	Carboplatin, Paclitaxel	Pembrolizumab (Anti PD-L1/PD-1)	Anti-tumor immune stimulation	II	NCT04964960
	Non-squamous NSCLC	Nab-paclitaxel	Cabozantinib S-malate, Docetaxel, Paclitaxel, Gemcitabine Hydrochloride	Nivolumab (Anti-CTL-4), Ramucirumab (Anti- VEGFR-2)	Anti-tumor immune stimulation	II	NCT04310007
	Thyroid carcinomas	Nab-paclitaxel	Cobimetinib, Vemurafenib, Paclitaxel	Atezolizumab (Anti PD-L1/PD-1), Bevacizumab (Anti-VEGF), Cobimetinib (MAPK inhibitor)	Anti-tumor immune stimulation	II	NCT03181100
	Metastatic triple negative breast cancer	Nab-paclitaxel	Carboplatin, Gemcitabine Hydrochloride, Poly ICLC	Durvalumab (Anti PD-L1/PD-1), Tremelimumab (Anti-CTL-4)	Anti-tumor immune stimulation	II	NCT03606967
OncoTherad® (MRB-CFI-1)	Non-muscle invasive bladder cancer (NMIBC)	Nanometric components formed by phosphate and metal salts (CFI-1)		Glycosidic proteins (P14 and P16 proteins)	Immunogenic cell death	I/II	Alonso et al.^97^

#### Target and delivery using cell therapies and nanoformulations

The first attempts to use immune cells to deliver nanoformulations was the “backpacking” approach to achieve T cell expansion by conjugating antigen loaded NPs to the outer membrane of T cells to deliver their contents (for example, IL-15 superagonist, which is IL-15/IL-15Rα complex, stimulates neighboring or opposite IL-15Rβ/γC-expressing cells via *trans*-presentation^101^) predominantly to the T cell in lymph nodes in a pseudo-autocrine way. This innovative technique was shown to reduce *in vivo* tumor toxicity and increases delivery selectivity by exclusively conjugating nanomaterials to the therapeutic T cell population. An example of such *backpacking* nanomaterials is the controlled-release lipid nanocapsules (NCs) loaded with SN-38, a strong topoisomerase I inhibitor, in a mouse model of Burkitt’s lymphoma. These NCs, generated by phosphatidylglycerol and maleimide-headgroup lipids, were covalently attached to the thiol groups on plasma membranes of *in vitro*-activated primary T lymphocytes cultured to promote the retention of CD62L and CCR7 receptor expression, both of which are essential for lymph node homing. T cells conjugated to SN-38-loaded NCs were deployed as live vectors to systemically transfer NCs into lymphoid tissues abundant with lymphoma cells. In an aggressive transplanted Eμ-*myc* Arf^−/−^ mice model (Burkitt’s lymphoma), where Eμ regulates the overexpression of c-*myc* and knockout of *Arf* leads to p53 inactivation, T cells functionalized with SN-38 NCs rapidly reduced tumor burden in several anatomical regions and dramatically extended survival compared to systemic chemotherapy. These findings imply that autologous lymphocytes with modified tissue-specific homing receptors can serve as effective chaperones for systemic cancer medication delivery.^102^ In another research, the strategy to backpack sorafenib-loaded lipid nanoparticles into M1 type macrophage was developed by Teng Hou and collaborators showing the synergistic effect: Sorafenib, a multityrosine kinase inhibitor approved by FDA for the treatment of hepatocellular carcinoma, functions as a cell proliferation blocker to directly kill tumor cells; meanwhile the targetability and delivery were enhanced by M1 macrophage.^103^

In another study, it was discovered that T cells can regulate their activation by modulating cell surface redox reactions particularly with the presence of cell surface free thiols. Researchers synthesized protein nanogels (NGs) carrying proteins such as a new IL-15 superagonist (IL-15-Sa—a fusion molecule between a mutant of IL-15, 15N72D, that has increased binding capabilities, and the IL-RaSu domain with the human IgG1 Fc domain),^104^ anti-CD45 or IL-2Fc (a fusion protein contain IL-2 fused with an antibody Fc fragment) and coupled them to T cells to target CD2, CD8, CD11α, and CD90 expressing melanoma cells *in vitro* and *in vivo*. The attachment to cell membranes was achieved via the NHS-SS-NHS crosslinker, which was fluorescently labeled with Alexa Fluor 647 NHS ester and biotinylated, and then was covalently attached to the plasma membrane of CD8^+^ T cells. Upon T cell activation through antigen encounter, e.g., in the TME, the disruption of crosslinkers in nanogels due to redox activity led to a cytokine release. The nanogels showed selective expansion of T cells up to 16-fold greater in tumors while remaining mostly dormant in peripheral blood. Additionally, there was an 8-fold higher release of cytokines leading to enhanced tumor clearance compared to free cytokine administration.^105^

The use of nano-therapies to assist the immune targeting abilities has been suggested for some time. At first by using liposomes targeting T cells, via an antibody against CD3, in this manner that the liposomes deliver ROS-scavenging molecules to T cells protecting them from oxidation-induced loss of activity,^106^ or by creating membrane nanoparticles from cells over-expressing antigens, and packed with adjuvants, to stimulate T cell responses against acute myeloid leukemia cells *in vivo.*^107^ Thus, the concept that nanomaterials and cellular therapies can complement each other is the very approach we advocate for. And while this concept is currently in its infancy, there are examples showing its potential. Yun Chang et al. designed and screened four chlorotoxin (CLTX)-CAR (T-specific CD3ζ or neutrophil-specific CD32a) constructs to compare their anti-tumor activity with neutrophil-specific signaling domains for targeting glioblastoma multiforme (GBM). These constructs were integrated into the adeno-associated virus S1 (AAVS1) safe harbor locus of human pluripotent stem cells (hPSCs) using CRISPR-Cas9-mediated homologous recombination. To facilitate targeted drug delivery, a biodegradable mesoporous organic silica NP with a rough surface (R-SiO2) was synthesized and utilized to load the hypoxia-activated pro-drug tirapazamine (TPZ), clinical chemo-drug temozolomide (TMZ), and a potent PRMT5 inhibitor JNJ-64619187. CAR-neutrophils loaded with drug-containing R-SiO2 NPs displayed superior anti-tumor activities against GBM, attributed to CAR-enhanced direct cytolysis and chemotherapeutic-mediated tumor-killing via cellular uptake and glutathione-induced degradation of NPs within targeted tumor cells.^108^

We suggest a 2-step approach, where *targeting* is followed by *delivery*, only in a sequential manner they actually work. We can draw conclusions that this concept is feasible from other elegant approaches such as the use of bacteria to deliver synthetic antigens to the TME, effectively tagging tumor cells for CAR-T cells engineered to target such antigens. Using this approach, named probiotic-guided CAR-T cells (ProCARs), researchers showed improved CAR-T efficiency in various human tumor xenografts and mouse syngeneic models.^109^ ProCARs combine synthetic CAR targets with probiotics that can colonize immune-protected tumor cores without relying on tumor-associated antigens (TAA); it is unclear how bacteria could be used in clinical therapy, the hurdles to reach that point are rather more complicated than those for NPs. Previously, engineered bacteria have also been employed in immunotherapy to improve antitumor immunity.^110^ In a study, a non-pathogenic *E. coli* strain Nissle (*EcN*, a safe probiotic bacterium, named after the researcher who described it^111^) was engineered to express IFN, therefore activating APCs cells and innate immune pathways via the complement system. Similarly, Thomas Savage et al. introduced *EcN* expressing combined CXCL16 and CCL20 to recruit activated T cells hence activating the immune system.^112^ The preferential accumulation of anaerobic bacteria in the TME compared to normal tissues, due to quorum sensing of hypoxic conditions, has been tested as a natural platform for the development of engineered cancer therapies.^113^ Additionally, other prokaryotes such as *Listeria* sp. have been shown to accumulate in heavily immune-suppressed tissues, such as tumors or metastases, while cleared from healthy tissues.^114^ This suggests that microbes, having motility and sensing, can target tissues specifically, and could also deliver metabolic cargos, but most importantly to attract the immune system to act locally.^113^

Most recent, Chenya Wang and collaborators drew inspiration from findings that Mn^2+^ functions as an agonist for the cGAS-STING pathway (cyclic guanosine monophosphate-adenosine monophosphate synthase-stimulator of interferon genes). This pathway is shown to play an important role in tumor suppression through immune checkpoint blockades (PD-1, PD-L1).^115^ Therefore, they developed MnO_2_ nanostructures that can be degraded into Mn^2+^, which coordinates with cyclic dinucleotide to grow on the surface of inactivated paraformaldehyde-fixed *Salmonella typhimurium*. This platform was then injected intratumorally to Balb/c, C57BL mice, and New Zealand white rabbits to activate the STING pathway, showing significantly tumor suppression activity.^116^ Yet, we could think that T cells or macrophages can do the very same upon genetic engineering, and more if combined with nanomaterials for the delivery of potent tumor-killing compounds.

As the TME is the main influencer of the polarization of TAMs to the M2 phenotype, an important driver for an immunosuppressive TME. In turn, M2 TAMs secrete immunosuppressive molecules, among them indoleamine 2,3-dioxygenase 1 (IDO1) which catabolizes tryptophan to kynurenine, increasing immunotolerance by consolidating the M2 phenotype and the expansion Treg. Using outer membrane vesicles (OMVs) derived from FimH-positive *E.coli*, nano-spherical bilayer of the membrane vesicles that are naturally secreted and retain the bacterial membrane/periplasmic composition, as these nanoscale vesicles enter macrophages via caveolin endocytosis – what gives them stability by avoiding lysosome processing,^117^ the authors wrapped these membranes with C16-ceramide and filled them in with R848 (a TLR7/TLR8 agonist) and INCB024360 (an inhibitor of IDO1). These OMVs where then loaded to glypican-3-targeting macrophages, a significant molecule in melanoma and hepatocellular carcinoma, and used to treat mice bearing H22 hepatocellular carcinomas.^118^ C16-ceramide induced the generation of OMV-containing exosomes in macrophages, which were then released upon macrophage detection of glypican-3 on H22 tumors in mice. The local secretion of R848 and INCB0244360 resulted in killing tumor cells and reversing the immunosuppressive TME.^118^

The advantages of nanomaterials lie in their biodegradability and inability to become pathogenic, something that probiotics are not, but all these examples indicate that the concept of *targeting and delivering* using a 2-step approach is possible and potentially far more efficient.

A different approach, in which exosomes were used or fused with NPs, has found that they exhibit targeting and delivery, acquiring the advantages of T cells or macrophages, and the delivery of nano-formulations. For example, exosomes derived from immune cells, crucial in cell-cell communications to transport molecules and signals to recipient cells via exocytosis and endocytosis. Using M1 macrophages as the source of exosomes, these exosomes were then injected into mice in combination with a mannose-functionalized lipid calcium phosphate (LCP) nanoparticulate vaccine. The M1 exosomes enhanced the uptake of NPs by dendritic cells via mannose receptors therefore LCP NPs encapsulated tyrosinase-related protein 2 peptides were released, stimulating dendritic cells or macrophages to produce Th1 cytokines and strengthening the cytotoxic T cell response to the antigen.^119^ Exosomes derived from T cells, especially those from CAR-T cells, can also play the same role. In a report by Pengxiang Yang et al. focusing on triple-negative breast cancer, exosomes derived from mesothelin-specific CAR-T cells exhibited antitumor activity through the secretion of perforin and granzyme B, effectively inhibiting tumor growth.^120^ NExT, introduced recently as a breakthrough of biomimetic nanoparticles (BNPs), made of a non-toxic poly(lactic-co-glycolic acid) (PLGA) coated with patient-derived exhausted T (ExT) cell membrane to synergize nanotherapy delivery with immune targeting of stromal PD/L1+ breast tumors.^121^ T cells from triple negative breast cancer patients were enriched *in vitro* and activated to express PD1, lymphocyte-activation gene 3 (LAG3), mucin-domain containing-3 (TIM3) immune checkpoint receptors then cell membranes were isolated and mixed with NPs. The NExT platform showed the enhanced interaction with tumor cells through corresponding ligands including PD-L1, Gelectin-3, protein fibrinogen-like protein 1, Gelectin-9, and Ceacam-1, thereby improving active targeting and delivery.^121^^,^^122^

### Future perspective for *target and delivery* by nano-cellular therapy

**Figure 6:**
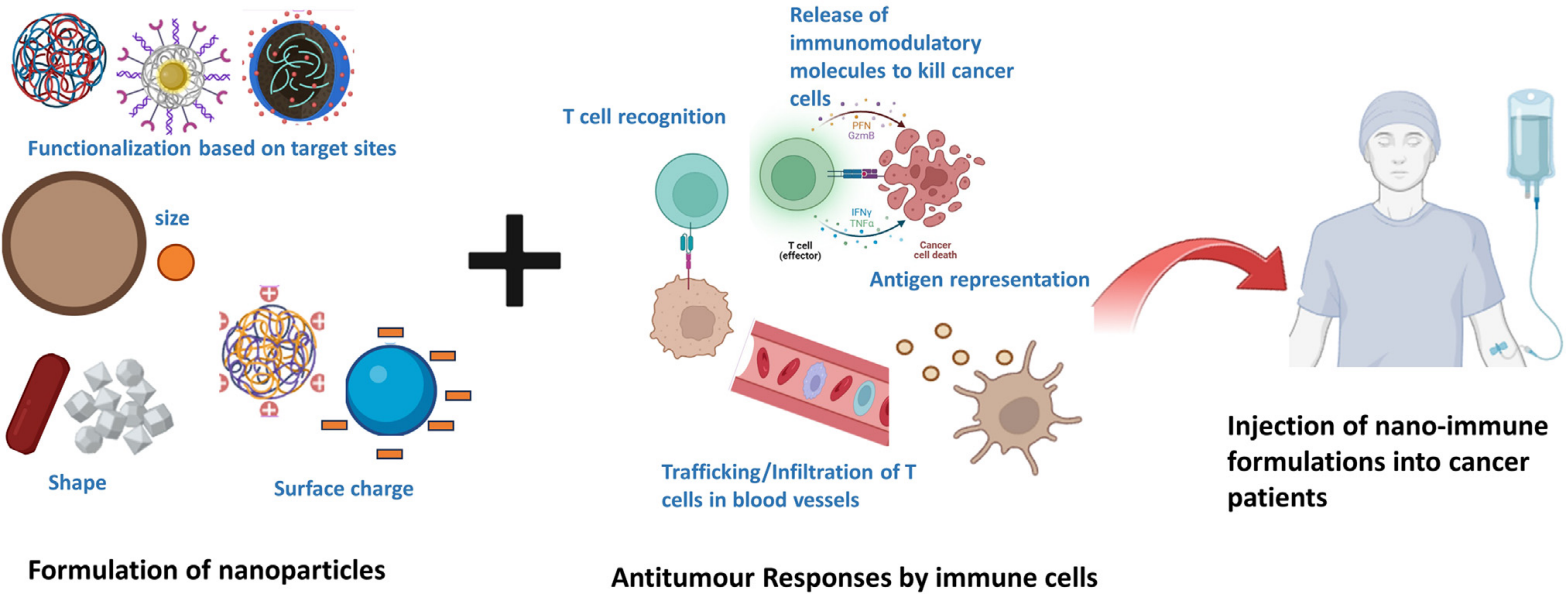
Tailoring the physicochemical and biological properties of nanoparticles to enhance the anti-tumor responses of immune cells is a key strategy in optimizing nano-immunotherapy Nano-immunotherapy harnesses the unique properties of nanoparticles to revolutionize cancer treatment by enhancing the body’s natural defenses. Nanoparticles, engineered with specific sizes, shapes, and surface charges, are functionalized with targeting ligands to selectively deliver immunomodulatory molecules to the TME. These tailored nanoparticles can stimulate anti-tumor immune responses by facilitating T cell recognition of cancer antigens, promoting the trafficking, and infiltration of immune cells into tumor sites, and enhancing antigen presentation. Ultimately, the release of immunomodulatory cargo from these nanoparticles aims to eliminate cancer cells, offering a targeted and potentially less toxic approach to immunotherapy.^123^^,^^124^^,^^125^

To address the challenges encountered in integrating NPs with immunomodulatory cells, it is important to understand the unique attributes of NPs like the encapsulation or immobilization of therapeutic payloads, interactions with serum proteins and cell surfaces, and the ability to penetrate anatomical blockades (Figure 6). The interaction of therapeutic cargos functionalized in NPs with immune cells like T cells or macrophages is yet another aspect to explore. These interactions could be manipulated according to the downstream signaling/metabolic pathways.

**Figure 7:**
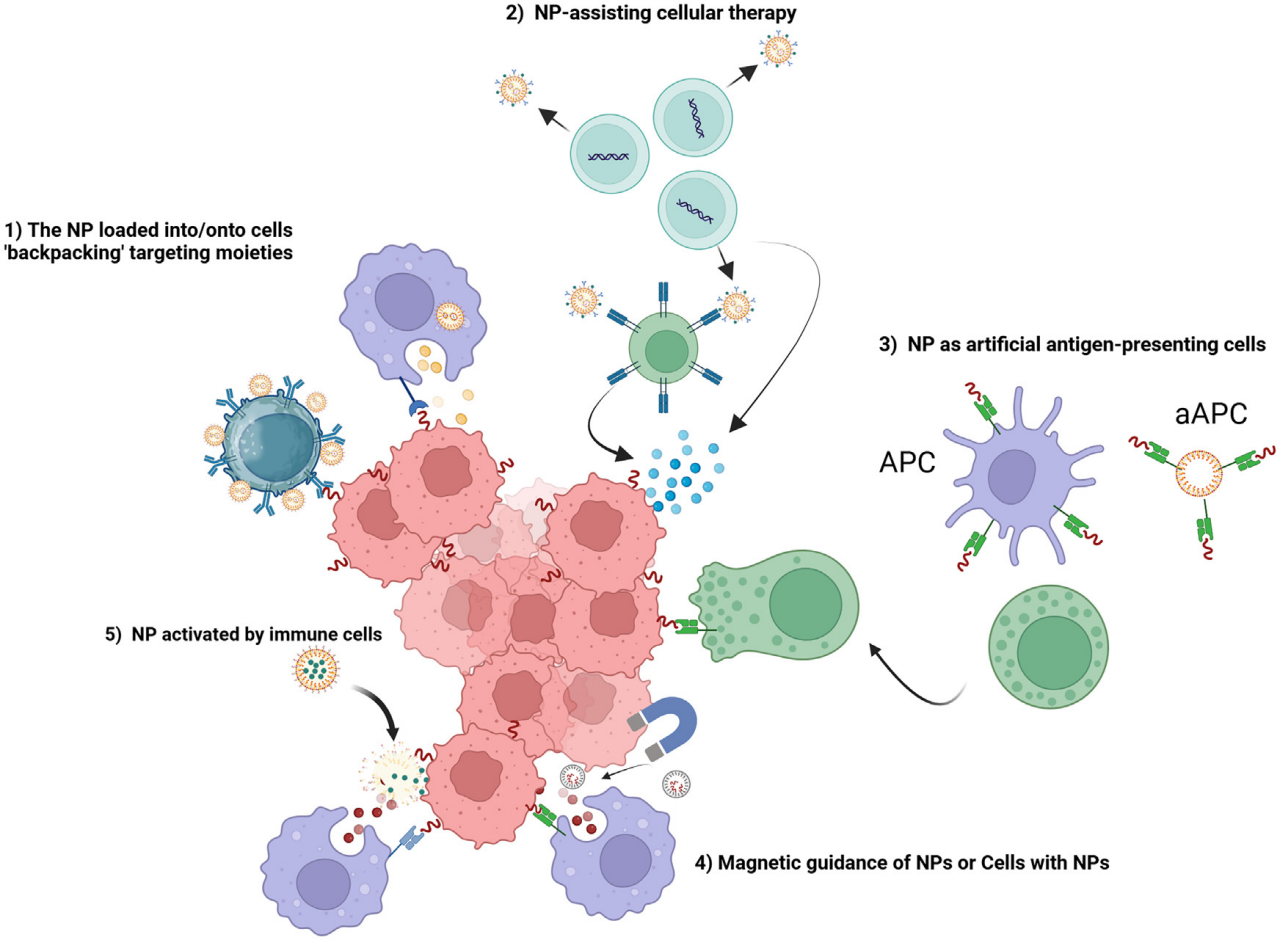
Considerations for trials involving the delivery of NPs to TME or to cancer cells (1) The backpacking of NPs into or onto immune cells may require some additional challenges; (2) NPs can also monitor the proliferation and differentiation of stem cells; (3) NPs-based aAPCs can enhance T cell activation and proliferation; (4) A magnetic nanocluster can be conjugated with T cells and improve the penetration of immune cells into tumor; (5) NPs can be presented in an inactivate form and then activated by immune cells.

In Figure 7, we present five essential considerations that must be taken into account when designing the testing system for either delivering NPs to the TME or delivering of functionalized NPs to tumor cells:

To-date, NPs have excelled in delivering molecules to cells; however, their targetability is questionable as the EPR effect might play a larger role than claimed for. Yet, they might pave the way for priming, assisting and/or activating cellular immunotherapies. Evidence suggests that enhanced T cell activation and proliferation can be achieved through utilizing aAPCs or NP-based T cell scaffolds prior to adoptive transfer. The potential of aAPCs to target the heterogeneous TME can be augmented by employing multiple antigens, promoting a comprehensive T cell response.^126^ Techniques using magnetic resonance imaging (MRI) guidance and magnetic nanocluster-conjugated T cells into tumors may also be relevant. In this scenario, a magnetic field might be employed to direct or locally activate tumor-specific T cells conjugated with magnetic NPs, addressing the challenge of T cell tumor penetration.^127^

As mentioned earlier, the backpacking of NPs onto immune cells or being carried as payloads by the cells may have some potential risks such as self-delivery. Ensuring the stabilization of nanomaterials inside or on immune cells may be challenging, particularly concerning toxicity, and the delivery of internally loaded NP cargos also requires additional molecular or chemical engineering for efficient active delivery. Although, some examples of “activatable” systems exist as presented previously. The interference of NPs with the functionality of these immune cells may also arise as another challenge that can be addressed by thoroughly examining the hurdles individually experienced by nanomaterials and cellular therapies.

One way to imagine nano-immuno-cooperation would be to use NP (or their cargo) in an inactive form, where activation e.g., by a non-endogenous/artificial enzyme is required. Such enzyme would be produced/delivered locally by the cellular therapy only upon target engagement. Such 2-step approach might be developed faster than NP-immune cell fusions as each technology separately has already been tested in multiple settings. A 2-step system is not unnatural, it was Paul Ehrlich himself who identified the “complement”—a second component that followed antibody binding and induce cell death (named *toxophoric group*).^128^

As our understanding of the TME and protein engineering advances, the complexity of potential targeting and delivering methods will also increase. Moreover, as our ability to control cell signaling/metabolism improves, thus, is the generation of more complex systems, systems that run under logic gates, feedback mechanisms and are more tightly regulated, allowing a better integration between chemistry and biology. We believe that any new therapeutic modality shall have the targeting ability of cellular therapies and the delivery potential of NP, whether this could be possible using a single new technology or would require 2-step approach, that is something that time, and a lot more research, will tell.

## Data and code availability

All data can be obtained from the corresponding author.

## Acknowledgments

Authors would like to thank the Polish National Science Centre (NCN) grant UMO-2020/39/I/ST5/03560 for the financial support. BioRender software was used for the production of the figures.

## Author contributions

T.H.N. and S.M. drafted the MS; A.R.-M. conceptualized the work; T.H.N. created all the figures.

## Declaration of interests

The authors declare no competing interests.
